# Synchronized Analysis of FTIR Spectra and GCMS Chromatograms for Evaluation of the Thermally Degraded Vegetable Oils

**DOI:** 10.1155/2014/271970

**Published:** 2014-01-19

**Authors:** Siong Fong Sim, Terri Zhuan Ean Lee, Nurul Aida Lu Mohd Irwan Lu, Benedict Samling

**Affiliations:** Universiti Malaysia Sarawak, Faculty of Resource Science and Technology, 94300 Kota Samarahan, Sarawak, Malaysia

## Abstract

Fourier Transform Infrared (FTIR) and Gas Chromatography Mass Spectrometry (GCMS) are two common instruments used for analysis of edible oils. The output signal is often analysed on the software attached to the workstations. The processing software is usually individualised for a specific source. The output of GCMS cannot be analysed on the FTIR hence analysts often need to juggle between instruments when multiple techniques are employed. This could become exhaustive when a large dataset is involved. This paper reports a synchronised approach for analysis of signal from FTIR and GCMS. The algorithm is demonstrated on a dataset of edible oils to investigate the thermal degradation of seven types of edible oils treated at 100°C and 150°C. The synchronised routines identify peaks present in FTIR and GCMS spectra/chromatograms where the information is subsequently extracted onto peak tables for further analysis. In this study, it is found that palm based products and corn oils were relatively more stable with higher content of antioxidants tocopherols and squalene. As a conclusion, this approach allows simultaneous analysis of signal from multiple sources and samples enhancing the efficiency of the signal processing process.

## 1. Introduction

Fourier Transform Infrared (FTIR) and Gas Chromatography-Mass Spectrometry (GCMS) are two essential techniques applied for analysis of edible oils [1, 2]. Fundamentally, FTIR spectra illustrate absorption bands with characteristic frequency attributed to different functional groups whilst GCMS reveals the compounds eluted at different retention times with mass spectra corresponding to compounds present, indicative of the fatty acid compositions. Conventionally the resultant signals from both instruments are analysed with the software equipped at the workstations for peak integration. The software is typically instrumental and model dependent. The signal processing tool exclusively designed for FTIR is not applicable to GCMS chromatograms due to differences in data nature and characteristics. Therefore to analyse the output from both FTIR and GCMS, an analyst has to juggle between both instruments. When a large volume of sample is involved, the signal processing process can be exhaustive and time consuming.

With the advances in computer technology, various alternatives have been made available reducing the dependence on the default signal processing tool; for instance, the digital data in csv format is readable on Microsoft Excel. Numerous algorithms have been developed for analysis of signal from various instrumentation techniques, that is, Fourier Transform Infrared (FTIR) [1, 3], Gas Chrnomatography-Differential Mobility Spectrometry (GC-DMS) [4], Gas Chromatography-Mass Spectrometry (GC-MS) [5–7], High Performance Liquid Chromatography (HPLC) [8], Nuclear Magnetic Resonance (NMR) [9], two-dimensional Gas Chromatography (GC-GC) [10], and Liquid Chromatography-Mass Spectrometry (LC-MS) [11] to allow mathematical integration of the signals including baseline correction, smoothing, and peak deconvolution on a personal computer. These algorithms however are designed to cater a specific source of signal; hence, when multiple sources of signals are involved more than one algorithm is required.

In edible oil industry, continuous monitoring and extensive cross comparisons between products involving multiple instrumental techniques such as FTIR and GCMS are common and this often results in enormous amount of data that requires a more efficient way for data interpretation. This has motivated the development of a signal processing approach that aids in analysis of signals from multiple sources. In this paper, we report a synchronised signal processing algorithm that caters both FTIR and GCMS signal for evaluation of thermally degraded vegetable oils.

## 2. Materials and Methods

### 2.1. Samples

Seven types of edible oils were studied including (1) palm oil, (2) canola and palm oil, (3) corn oil, (4) canola oil, (5) soybean oil, (6) blended palm, sesame and peanut oil, and (7) canola and sunflower oil. They were obtained from the local market and heated at 100°C and 150°C, respectively, for 15 min using a digital heating block. The oil samples were then left to cool and analysed using FTIR and GCMS. A total of 63 FTIR spectra and GCMS chromatograms, respectively, were produced (7 types × 3 treatments × 3 replicates = 63).

### 2.2. Fourier Transform Infrared (FTIR)

All spectra were obtained using an ATR-FTIR of ThermoScientific (Thermo Nicolet Analytical Instruments, Madison, WI). The spectra were collected at a resolution of 4 cm^−1^ in the range of 4000–650 cm^−1^. Each spectrum was rationed against a fresh background spectrum recorded from the bare ATR crystal. Prior to collection of each background spectrum, the ATR crystal was cleaned with absolute ethanol to remove any residual. Each sample was scanned in triplicate.

### 2.3. Gas Chromatography-Mass Spectrometry (GC-MS)

For GCMS analysis, 100 *μ*L of oil was dissolved in 2 mL of dichloromethane (DCM). The sample was analysed on a Shimadzu GC-MS system model QP500 with a medium polarity capillary column (BPX-5 column (29.4 m × 0.25 mm), with film thickness of 0.25 *μ*m) with helium as the carrier gas. One microlitre of the sample was injected using splitless injection with injector temperature 300°C according to the following scheme: 50°C for 2 min with 10°C/min up to 300°C. The final temperature was held for 10 min. The total runtime for each sample was 37 min. For MS detection, electron ionization with 70 eV was applied and mass fragments were detected between 40 and 500 *m/z*. The ion source temperature and transfer line temperature were 200°C and 300°C, respectively. Note that the detector was activated after 5 min.

### 2.4. Signal Processing

The FTIR spectra and GCMS chromatograms were analysed using the synchronised algorithm developed in Matlab R2012a. The synchronised strategy is an extension of the peak detection and matching algorithm designed for FTIR, published in [12]. First, all data files (csv format for FTIR spectra and netcdf for GCMS chromatograms) were converted into mat files. The algorithm requires input of the data nature whether it is one-dimensional or two-dimensional. If the data is two dimensional, the algorithm would obtain the total ion chromatogram (summing the intensities of all mass spectral of the same scan) compressing them into one-dimensional data. The data is subsequently baseline corrected according to asymmetric least squares [13], smoothed using soft heuristic thresholding (*sym8* wavelet) [14], and transformed into first derivative signal where the peak start, peak, and peak end are identified. Briefly, the algorithm would evaluate each data point of the derivative signal in succession; a peak start is labelled when the derivative signal is above zero and *x* times greater than the peak noise (average absolute change of derivative); when the signal crosses *x*-axis attaining negative values, the peak is located. As the derivative signal crosses *x*-axis again where the values become positive, peak end has arrived [12]. Upon detection of a peak, the corresponding peak area is calculated as the sum of the detector output between the start and the end. The algorithm would evaluate the spectrum in turn to identify the peaks present; they will then be matched across samples for similar functional groups according to a predefined window size. For example, if a window of *z*_1_ scans is set and a peak at 1720 cm^−1^ is targeted for matching, the algorithm would search through all samples for possible matching peaks ranging between 1720 ± *z*_1_ cm^−1^. The matching peaks are subsequently arranged in the same column with rows corresponding to samples; as a result, a table representative of peaks detected and matched is produced. It is important to evaluate the resultant table to confirm that parameters employed such as window size and peak threshold are adequate. If a potential matching peak is mismatched or unmatched, it is an indication that the window size would require fine-tuning. In terms of peak detection, it is possible that the algorithm suffers to identify poorly resolved bands or shoulder bands; sometime the peak noise threshold may be unsuitable, too small that noise is misidentified as signals or too large that signals are overlooked. Therefore, it is essential to manually verify the information on the peak table overlaying the spectra for visualization.

**Figure 1:**
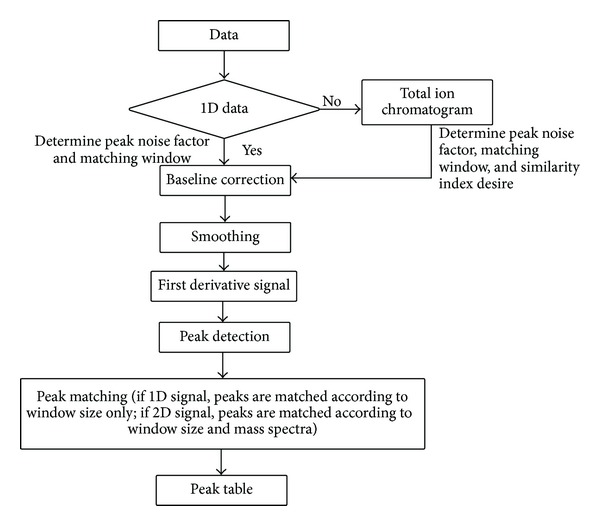
Schematic diagram of the synchronised algorithm.

For GCMS data, peaks are detected similarly based on the total ion chromatograms; however, in the matching process, the candidate matching peaks are identified according to a predefined window size, *z*_2_ (in this paper, *z*_2_ = ±50 scans (±15 s)), and further confirmed with the mass spectra according to the similarity index. Spectra with correlation coefficient >90% are considered corresponding to the same compound in which matching peaks are organised into a peak table as described above. For each compound identified, the mass spectrum is recorded. Prior to peak matching, the spectra were prealigned to some common peaks according to the strategy of retention time alignment in [15]. In oil analysis, prealignment, and setting, an optimum window size is crucial to minimize erroneous matching as compounds eluted closely may exhibit mass spectra with high similarity, sometime more than 98% for example, 2,4-dodecadienal and 2,4-decadienal eluting at 11.98 min and 12.35 min, respectively.  Figure 1 shows the schematic diagram of the synchronised algorithm. Two peak tables were produced resulting from the analysis of FTIR and GCMS spectra/chromatograms. The algorithm undoubtedly experiences some inherited shortcomings; nevertheless, it enables multiple output signals from analytical instruments to be processed efficiently and systematically. The peak table was preprocessed (square rooted, scaled to one, and standardised) and subjected to Principal Component Analysis (PCA) for further analysis.

## 3. Results and Discussion

**Figure 2:**
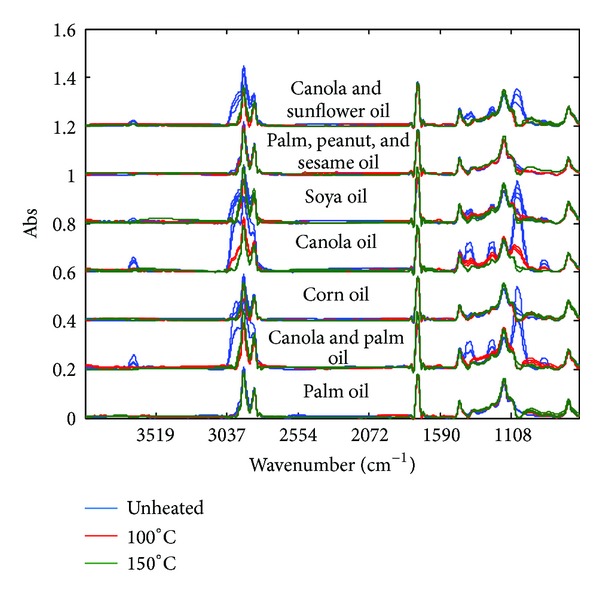
The FTIR spectra of seven edible oils treated under different temperatures, unheated, 100°C and 150°C.

**Figure 3:**
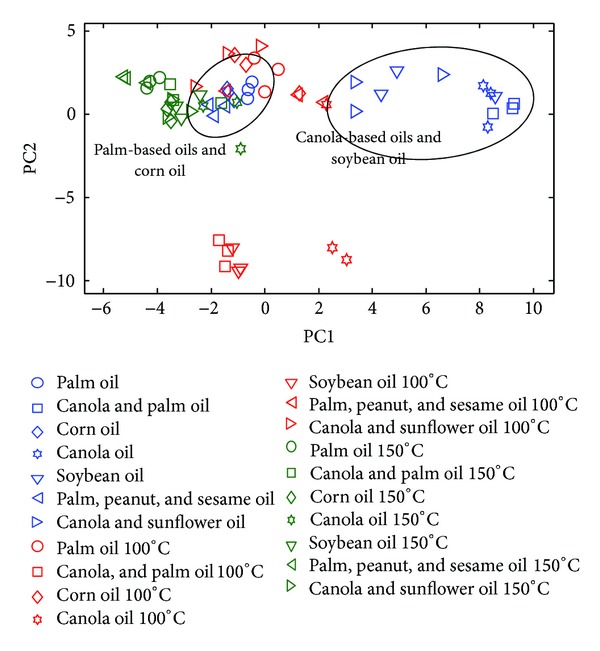
The scores plot of PC2 versus PC1 of the peak table obtained from FTIR analysis.

The algorithm yields two peak tables corresponding to the analysis of a total of 63 FTIR and GCMS spectra/chromatograms, respectively.  Figure 2 shows the FTIR spectra of seven edible oils treated under different temperatures: unheated, 100°C, and 150°C. Essentially the superimposed spectra of heated and unheated palm oil, corn oil, and blended palm oil exhibit slight variation suggesting little changes upon treatments. The scores plot of the FTIR peak table in Figure 3 shows that canola-based oils are differentiable from palm-based oil. After heating at 150°C, various vegetable oils regardless of the origin are observed to cluster implying sharing of common features where palm oil encountered comparatively less alteration, inferring better thermal stability.

**Figure 4:**
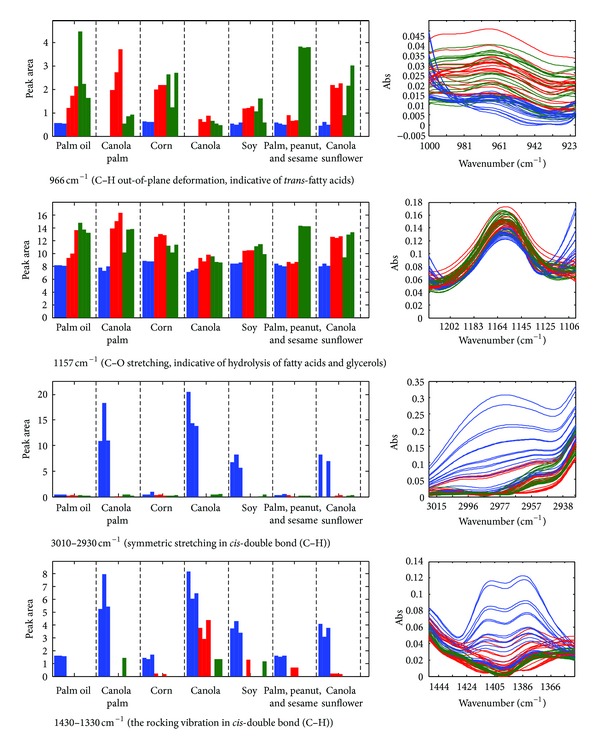
The relative abundance of some discriminatory bands of FTIR spectra with blue, red, and green bars indicating unheated samples and samples heated at 100°C and 150°C, respectively.

Typically, heated cooking oils are challenged with the loss of unsaturation due to the attack of oxygen *via* radical reaction [16]. The degree of unsaturation is often monitored based on several characteristic bands at 1650 cm^−1^ (C=C stretching vibration of *cis*-olefins), 1417 cm^−1^ (rocking vibrations of CH bonds of *cis*-disubstituted), and 3001 cm^−1^ (CH stretching vibration of the *cis*-double bond) [17]. If only a limited number of spectra are involved, there is no issue related to identification of changes due to thermal treatment; however, the process can be exhaustive when numerous spectra are concerned. The algorithm allows efficient evaluation of a large number of samples.  Figure 4 illustrates the relative abundance of some bands commonly reported for discrimination of thermally degraded oil. Evidently, the band corresponding to olefinic attribute at 1430–1330 cm^−1^ reduces steadily/disappears upon heating confirming the loss of double bonds where palm oils are characterised by relatively less polyunsaturated compounds, which is not unexpected. Other lines of evidence of diminished unsaturation are observed at 3010 cm^−1^ and 1650 cm^−1^. As oxidation continues, the degree of unsaturation is correspondingly reduced closing the gap of difference between polyunsaturated and polysaturated oils, leading to clustering of the thermally oxidised edible oils as demonstrated in Figure 3. In addition to loss of unsaturation, elevated temperature simultaneously triggers hydrolysis of triglycerides yielding fatty acids and glycerols, evidenced with increased band intensity at 1157 cm^−1^ (C–O ester groups). The autooxidation of unsaturated fatty acid also leads to emission of volatile aldehydes. The band at around 1680 cm^−1^, indicative of the C=O stretching of conjugated unsaturated aldehydes, is profoundly detected in heated oils of which palm oil demonstrated greater abundance. Formation of *trans*-fatty acid, a common issue in thermally oxidised oil, is associated with the band near 966 cm^−1^; it is often identified in a small amount in fresh oil as a consequence of isomerisation of *cis*-unsaturated fatty acids upon bleaching, refining, and deodorization [18–20]. The undesirable conversion is witnessed to be encouraged under increasing temperature confirmed by soaring of the band at 966 cm^−1^ in which palm olein oil and blended canola-sunflower oil demonstrate higher concentration after heating. As suggested elsewhere, palm oil and sunflower oil are characterised by better efficiency of heat transfer, thus leading to higher conversion of *trans*-fatty acid [21, 22]. The information on the peak table is converted into bar charts that are verified by overlaying the spectral at a designated region as illustrated in Figure 4; this approach enables rapid validation of the algorithm.

**Figure 5:**
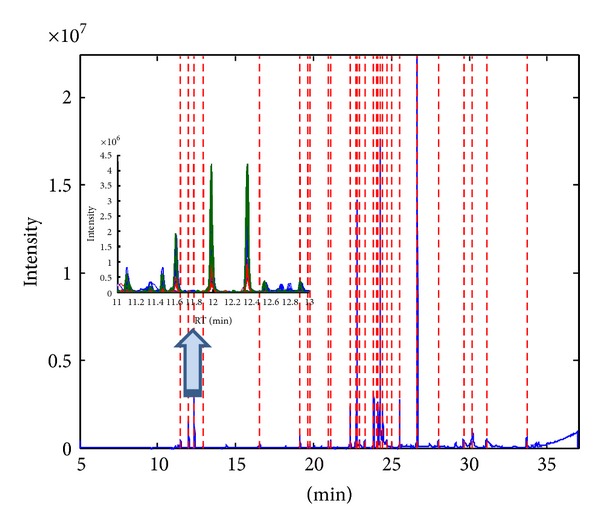
Peaks identified in a GCMS chromatogram with the inset exhibit several commonly present dienaldehyde peaks matched across the chromatograms with blue, red, and green lines indicating unheated samples, samples heated at 100°C and 150°C, respectively.

**Figure 6:**
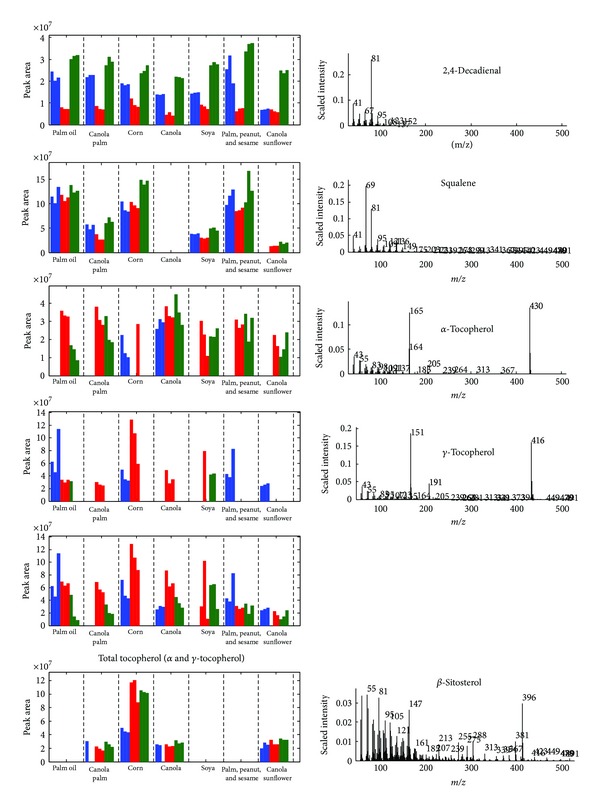
The relative abundance and mass spectra of several compounds identified in GCMS chromatograms.

The algorithm is designed for simultaneous analysis of GCMS chromatograms.  Figure 5 illustrates the peaks precisely identified in a chromatogram and the inset shows several dienaldehydes commonly present in all chromatograms. The information extracted is similarly translated into a peak table.  Figure 6 shows the relative abundance of the peak area corresponding to several compounds. Most cooking oils are enriched with vitamin E that exists in various forms mainly tocopherols and tocotrienols. Their presence essentially improves the antioxidation property of cooking oil as the compounds, during oxidation process, compete with unsaturated fats for lipid peroxy radical [23]. According to Al-Saqer et al. [24], these compounds are prominently found in soybean, canola, sunflower, and corn oils with relatively lower amount in palm oil. In this study, only *α*- and *γ*-tocopherols are identified; they are relatively more prominent in blended and pure palm oil as opposed to the findings of Al-Saqer et al. [24]. Nevertheless the nutritional label on the products, when compared, indicates that blended and pure palm oils possess higher vitamin E corresponding to the relative abundance of the total tocopherols revealed by GCMS: palm, peanut, and sesame (75 mg/100 mL); palm oil (60 mg/100 mL); corn (63 mg/100 mL); canola and palm (50 mg/100 mL); canola and sunflower (30.8 mg/100 mL); canola (20.3 mg/100 mL); soya (15 mg/100 mL). The higher concentration of vitamin E in palm based oil provides an explanation to the better oxidative stability suggested from the analysis of FTIR spectra. The various forms of tocopherols are in addition convertible; several studies have reported the conversion of *γ*- and *δ*-tocopherols into *α*- and *β*-tocopherols [25, 26]. As observed in this study, the *γ*-form of tocopherol is distinctively found in unheated palm-based oil, corn oil, and canola-sunflower oils; upon heating, this compound is completely missing and replaced with *α*-tocopherol that appears to degrade with increasing temperature.

Squalene is also a compound commonly found in vegetable oils, particularly high in olive oils, with antioxidant property. As illustrated in Figure 6, squalene is detected profoundly in blended and pure palm oil as well as corn oil corroborating the hypothesis drawn from FTIR spectra that these varieties of oils are more resistant to oxidation. Plant sterols including sitosterol, campesterol, and stigmasterol are cholesterol-like molecules; the saturated analogues are suggested as effective cholesterol-lowering agents whilst those with ethylidene contain side chain that may behave as antioxidants [27, 28]. In this study, *β*- and *γ*-sitosterols are identified with the former distinctively detected in canola and corn oils where the finding is in agreement with Ratnayake et al. [29].

During oxidation, fatty acids are typically converted into hydroperoxides and further broken down into secondary products such as aldehydes and ketones [30–32]. It is found that dienaldehydes such as 2,4-nonadienal and 2,4-decadienal are consistently detected in all oil samples where the concentrations appear to increase appreciably after heating at 150°C with no significant different concluded statistically (*p* > 0.05). Interestingly at 100°C, a decline in the amount of dienaldehydes is consistently experienced in all oil varieties, possibly due to the chain reaction of transformation to ultimate by-products.

## 4. Conclusion

The study demonstrates that output from FTIR and GCMS can be simultaneously analysed using a common signal processing approach for evaluation of thermally degraded vegetable oils. This would save the time for an analyst to juggle between instruments and applies some common guidelines to process the signals systematically. Undoubtedly, the approach is not a perfect and flawless method as there are possibilities that peaks are overlooked or mismatched; nevertheless, it offers a systematic strategy to process relatively large datasets of edible oils from GCMS and FTIR simultaneously with reasonable reliability.
